# Flavivirus Cross-Reactivity to Dengue Nonstructural Protein 1 Antigen Detection Assays

**DOI:** 10.3390/diagnostics10010011

**Published:** 2019-12-24

**Authors:** Li Kiang Tan, Wing Yan Wong, Hui Ting Yang, Roland G. Huber, Peter J. Bond, Lee Ching Ng, Sebastian Maurer-Stroh, Hapuarachchige Chanditha Hapuarachchi

**Affiliations:** 1 Environmental Health Institute, National Environment Agency, Singapore 38667, Singapore; 2 Bioinformatics Institute, Agency for Science, Technology and Research, Singapore 138671, Singapore; 3 Department of Biological Sciences, National University of Singapore, Singapore 117558, Singapore; 4 School of Biological Sciences, Nanyang Technological University, Singapore 639798, Singapore

**Keywords:** flavivirus, diagnostics, nonstructural protein 1, cross-reactivity, false positives

## Abstract

Dengue virus (DENV) and Zika virus (ZIKV) are flaviviruses of public health relevance. Both viruses circulate in the same endemic settings and acute infections generally manifest similar symptoms. This highlights the importance of accurate diagnosis for clinical management and outbreak control. One of the commonly used acute diagnostic markers for flaviviruses is nonstructural protein 1 (NS1). However, false positives due to antigenic cross-reactivity have been reported between DENV and ZIKV infections when using DENV NS1 antigen (NS1 Ag) detection assays in acute cases. Therefore, we investigated the lowest detectable virus titres and cross-reactivity of three commercial dengue NS1 Ag rapid assays and two ELISAs for different flaviviruses. Our results showed that substantially high viral titres of ZIKV, Kunjin virus (KUNV) and yellow fever virus (YFV) are required to give false-positive results when using DENV NS1 rapid detection assays. Commercial DENV NS1 ELISAs did not react with ZIKV and YFV. In comparison, tested assays detected DENV at a significantly low virus titre. Given the relatively low viral loads reported in clinical samples, our findings suggest that commercially available dengue NS1 Ag detection assays are less likely to generate false-positive results among clinical samples in areas where multiple flaviviruses cocirculate.

## 1. Introduction

Zika virus (ZIKV) is an emerging mosquito-borne virus that is closely related to other flaviviruses of public health relevance, such as dengue virus (DENV) and yellow fever virus (YFV). ZIKV infections in various African countries and Southeast Asia from the 1950s to the 1990s were largely unnoticed until epidemics occurred in the Pacific Islands after 2007 and in Latin American countries in 2015 [1,2]. ZIKV and DENV coexist in tropical and subtropical regions, where there are favourable environmental conditions for their main vector, *Aedes* spp. mosquitoes. Unlike many other flaviviruses, ZIKV is also known to be transmitted through sexual contact and perinatal transmission that is associated with potential consequences of congenital neurological malformations [2]. As such, the accurate diagnosis of ZIKV infections is essential for proper clinical management and public health risk assessment. Uncomplicated acute infections of ZIKV and DENV are difficult to be clinically differentiated. At present, confirmatory diagnosis of ZIKV infections largely relies on the detection of viral RNA either in sera or urine by polymerase chain reaction (PCR) [3]. On the other hand, besides PCR, the diagnosis of dengue is widely achieved by using dengue nonstructural 1 antigen (NS1 Ag)-based assays due to their ability to confirm the infection early and rapidly [4].

NS1 is a secretory glycoprotein generated during flavivirus replication. Upon infection, host cells synthesise NS1 as a soluble monomer. The protein rapidly homodimerises in the lumen of the endoplasmic reticulum (ER) and associates with the ER membrane. NS1 is transported through the secretory pathway to the cell surface and released into extracellular milieu [5] as a barrel-shaped hexamer containing a lipid cargo at its centre. Secreted NS1 (sNS1) exists stably as a hexamer in solution [6] and accumulates in serum in high amounts [7]. sNS1 is detectable in patient sera as early as the first day of fever and can last even up to nine days after fever onset [7,8]. Therefore, sNS1 can be used as a versatile marker for the early diagnosis of flavivirus infections [9,10,11,12]. However, commercial assays are available only for the detection of DENV NS1 Ag at present. The NS1 structural similarities among different flaviviruses could jeopardise the specificity of NS1-Ag-based assays, resulting in false-positive diagnostic results. Cross-reactivity between DENV and ZIKV when using a DENV NS1 Ag detection assay has previously been reported [13]. However, such observations are sporadic and have not been made in large patient cohorts [14]. Therefore, it still remains unclear whether commercial dengue NS1 Ag detection assays are appropriate to be used in endemic regions where other flaviviruses cocirculate.

In the present study, we therefore evaluated three DENV NS1-Ag-based rapid assays and two ELISAs for the possibility of cross-reactivity with ZIKV, YFV and Kunjin virus (KUNV), a surrogate for the West Nile virus (WNV). We determined the lowest virus titre detectable by each assay for different flaviviruses by measuring the virus titre and RNA copy numbers and mapped the NS1 amino acid differences of these flaviviruses to demonstrate the degree of NS1 antigen structural similarity that may contribute to cross-reactivity. Our findings showed that NS1 Ag assays designed for the diagnosis of dengue could generate false-positive results in ZIKV, KUNV and YFV infections but at substantially higher virus titres than those reported in natural infections due to respective viruses.

## 2. Materials and Methods

### 2.1. Cells and Virus Preparation

The viruses used in this study are summarised in Table 1. ZIKV, YFV and KUNV were propagated in the Vero cell line (ATCC© CCL-81™) at 37 °C for 3–5 days. Vero cells were maintained in M199 growth medium (Hyclon Laboratories, Logan, UT, USA) supplemented with 10% heat-inactivated foetal bovine serum (FBS) (Biowest, Riverside, MO, USA), 2 mM l-glutamine, 100 mM penicillin/streptomycin, 10 mM HEPES and 1 mM sodium pyruvate. DENV and chikungunya virus (CHIKV) were propagated in the *Aedes albopictus* cell line (C6/36, ATCC© CRL 1660™) and baby hamster kidney (BHK) cells, respectively, as previously described [15]. All viruses used in this study were passaged in the laboratory for no more than seven times. Maintenance medium used for viral infection and quantification was similarly prepared as above but supplemented with 3% FBS. CHIKV, an alphavirus, was used in the study as a negative control.

### 2.2. Viral Infection and Harvesting

To rule out the protein saturation effect (nonlinear serial dilution effect) [16] and to ensure that the titrated viral load was proportional to the accumulating amount of viral protein in culture, two independent time-course experiments were conducted. Cell cultures without viral infection were included as the infection negative control. The culture supernatants were collected for viral quantification and evaluation of NS1 Ag assays. Vero cells were seeded at a density of 1 × 10^5^ cells/mL in 24-well plates and infected at multiplicity of infection (MOI) of 0.1. After 1 h of adsorption, cells were washed with FBS-free medium before incubation in maintenance medium at 37 °C with 5% CO_2_. Culture supernatants were harvested at different time points postinfection after removing cell debris by centrifugation at 3000 rpm at room temperature. The harvesting was done at 0 h postinfection (h.p.i.), followed by intervals of every 6 and 12 h till 60 h.p.i. for DENV and ZIKV, respectively. The harvesting period was extended till 96 h.p.i. for KUNV and YFV. CHIKV cultures were harvested at 96 h.p.i. Harvested supernatants were stored in aliquots at −80 °C.

### 2.3. Quantification of Infectious Virus Particles and Viral RNA Copies

Culture supernatants of all virus isolates collected at each time point were quantified in triplicate for infective viral titres by plaque assay, as previously described [15]. In addition, the ZIKV and DENV RNA copies were quantitated in the same triplicate samples by using probe-based quantitative real-time PCR (qRT-PCR), as previously described [17,18].

### 2.4. Evaluation of Commercial Dengue NS1 Rapid Assays and ELISAs

Three dengue NS1-Ag-based rapid assays and two dengue NS1-Ag-based ELISAs were evaluated in this study. All are commercially available and widely used for the diagnosis of dengue. The rapid assays, designed based on lateral flow immunochromatographic technology, included SD BIOLINE Dengue NS1 Ag rapid test (Standard Diagnostics Inc, Geonggi-do, Republic of South Korea), Panbio Dengue Early Rapid (Alere Inc., Waltham, MA, USA) and Bio-Rad Dengue NS1 Ag STRIP (Bio-Rad, Marnes-la-Coquette, France). ELISAs included SD Dengue NS1 Ag ELISA (Standard Diagnostics Inc, Geonggi-docity, Republic of South Korea) and Panbio Dengue Early ELISA (Alere Inc., Waltham, MA, USA). The assays were selected based on the DENV detection sensitivity and specificity of more than 90% as claimed by the manufacturers or published in the literature. Tests were performed according to manufacturers’ instructions. For rapid assays, results were read at 15–30 min after the final step of each test. For ELISAs, the absorbance values were read with a microplate reader (Tecan Group Ltd., Mannedorf, Switzerland) at 560 nm within 30 min upon the addition of a stop buffer.

### 2.5. Sequence Analysis, Localization of Mutations and Prediction of Epitopes Targeted by Assays

The NS1 sequences of all viruses used in this study were aligned for pairwise differences using MAFFT [19], employing the L-ins-I local pairs strategy for maximum precision. Subsequently, mutations between all individual pairs were identified and projected on the surfaces of the full-length ZIKV NS1 structure (PDB 5K6K) [20]. The mutation maps were rendered using PyMol [21] with mutations indicated in red. All maps were then plotted in a matrix for pairwise comparison, with the lower and upper triangular sections representing a top and bottom view of the NS1 protein, respectively. Because the epitope regions targeted and antibodies used by each assay are proprietary, there was no direct approach to simulate the structural differences of NS1 epitopes targeted by different assays, which could explain the differential reactivity patterns observed for each assay. Instead, we predicted the epitopes likely engaged by each assay in silico by generating all potential epitopes as regions of 12A surrounding an individual residue as present in Zika NS1 structure PDB 5K6K. In total, 351 putative epitopes were generated and the sequences of all viruses for the respective epitope candidates were extracted from the sequence alignment. From these sequences, a pairwise similarity matrix for each candidate was calculated and compared with the similarity observed in assay reactivity. This allowed us to identify several epitope candidates, of which the pattern of sequence identity matched the similarity of assay reactivity. The overlapping epitope candidates were merged to identify the epitope regions potentially targeted by the evaluated assays.

### 2.6. Statistical Analysis

Student’s *t* test was used to compare viral titres and RNA copy numbers among different assays. Data are expressed as average at log base 10 ± standard deviation and considered significant when *p* < 0.05. The analyses were done using Excel 2010 software (Microsoft version 2010, Redmond, WA, USA).

## 3. Results

### 3.1. Dengue NS1 Ag Assays Detected DENV Early and at Low Titres

The virus titres in culture supernatants detected by DENV NS1 Ag rapid assays and ELISAs for DENV-1–4 were compared with other flaviviruses. The growth kinetics of all viruses showed a gradual increase in viral load before the virus titres hit the plateau (Figures S1–S3). ELISAs detected DENV at a lower titre (Table 2) and an earlier time point than rapid assays. ELISAs detected all DENV serotypes in culture supernatants as early as 24 h.p.i. On the other hand, the time of first detection for rapid assays varied among DENV serotypes (DENV-1: 42 h.p.i.; DENV-2 and -3: 36 h.p.i.; DENV-4: 48 h.p.i.). The lowest detectable titres for DENV were as low as 0 log plaque-forming unit (pfu)/mL (range: 0–4.22 log pfu/mL) (Table 2). The SD BIOLINE Dengue NS1 Ag rapid test detected DENV-1–3 at a significantly lower titre (3.07–3.59 log pfu/mL) than the Panbio Dengue Early Rapid assay (3.39–4.22 log pfu/mL; *p* ≤ 0.005). Similarly, the SD Dengue NS1 Ag ELISA detected DENV-1–4 at significantly lower titres (0–0.57 log pfu/mL) than the Panbio Dengue Early ELISA (2.20–3.25 log pfu/mL; *p* ≤ 0.04), with at least 1 log difference within each serotype. All assays generated negative results for uninfected cell controls.

### 3.2. SD BIOLINE Dengue NS1 Ag Rapid Test and Panbio Dengue Early Rapid Assay Showed False-Positive Results for ZIKV at Significantly Higher Titres than DENV

**Figure 1:**
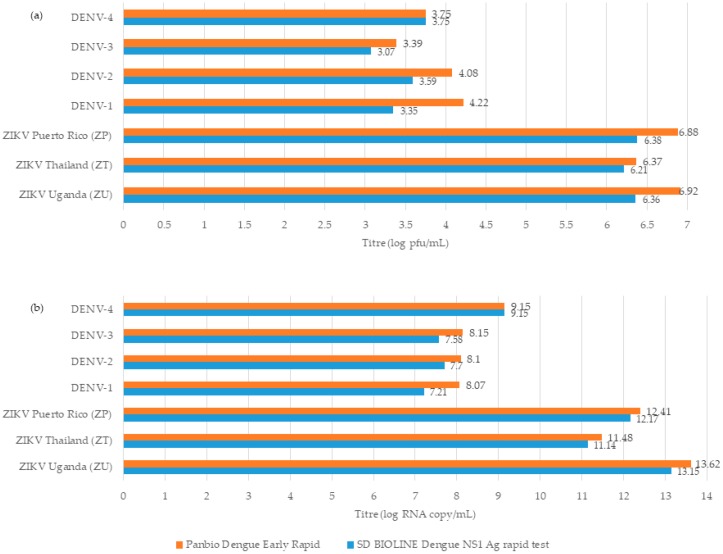
Lowest virus titres of DENV and ZIKV that gave positive results with SD BIOLINE Dengue NS1 Ag rapid test and Panbio Dengue Early Rapid assay. (**a**) Virus titres in log pfu/mL. (**b**) Virus load in log RNA copies/mL. Detailed data are provided in Table S1. pfu: plaque-forming unit.

SD BIOLINE Dengue NS1 Ag rapid test and Panbio Dengue Early Rapid assay gave false-positive results for all ZIKV strains in culture supernatants (Figure 1 and Table S1). The earliest time point of false detection was at 36 h.p.i. However, the lowest detectable ZIKV titres for both assays were significantly higher than those for DENV (6.21–6.92 log pfu/mL vs. 3.07–4.22 log pfu/mL, *p <* 0.02). Interestingly, both assays did not produce any false-positive results with any ZIKV strain at 24 h.p.i. (mean titre of log 5.3 pfu/mL) and 30 h.p.i. (mean titre of log 5.78 pfu/mL). Bio-Rad Dengue NS1 Ag STRIP rapid assay and two ELISAs did not show positivity to different ZIKV strains at any time points tested.

### 3.3. SD BIOLINE Dengue NS1 Ag Rapid Test and Panbio Dengue Early Rapid Assay Showed Mixed False-Positive Results for KUNV and YFV at Higher Titres than DENV

KUNV culture supernatants collected at 24 and 30 h.p.i. were positive for SD BIOLINE Dengue NS1 Ag and Panbio Dengue Early Rapid assays, respectively (Table 3). Both assays were negative at 12 h.p.i. (1.23 ± 1.08 log pfu/mL). However, the false detection was at a significantly higher KUNV titre (5.96 ± 0.23 log pfu/mL) compared with DENV-1–4 (3.07–4.22 log pfu/mL; *p* < 0.03). The Bio-Rad Dengue NS1 Ag STRIP assay remained nonreactive to KUNV culture supernatants even at 96 h.p.i. Interestingly, in contrast to other viruses, the Panbio ELISA also generated false-positive results for KUNV culture supernatants at a time point as early as 24 h.p.i.

On the other hand, only the SD BIOLINE Dengue NS1 Ag rapid test gave false-positive results with YFV culture supernatants but at a relatively late time point (96 h.p.i.). The assay was negative for YFV at 72 h.p.i. However, the lowest YFV titre falsely detected by the assay (7.14 ± 0.37 log pfu/mL) was significantly higher than that for DENV-1–4 (3.07–3.75 log pfu/mL; *p* = 0.002). The remaining assays were not positive for YFV. CHIKV culture supernatants collected at 96 h.p.i. served as a negative control and showed no positive results for any assay.

### 3.4. Similarity of Residues among Flavivirus NS1 Dimers Illustrated the Possibility of Cross-Reactivity

**Figure 2:**
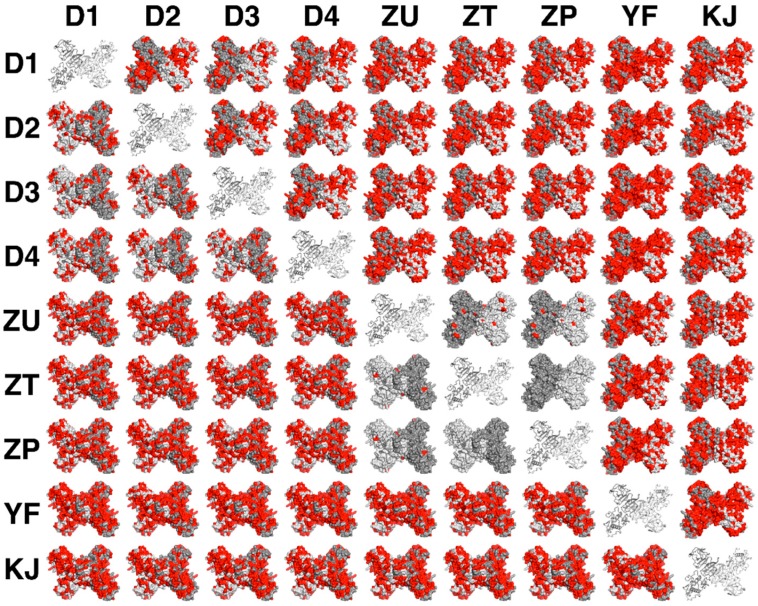
Pairwise differences visualised for the NS1 dimer. Sequences of DENV-1–4 (D1–D4); ZIKV Uganda strain (ZU), ZIKV Thailand strain (ZT) and ZIKV Puerto Rico strain (ZP); YFV (YF) and KUNV (KJ) were aligned using MAFFT [19]. The differences between all pairs were identified and mapped onto the recent high-resolution X-ray structure of the ZIKV NS1 dimer 5K6K [20]. Dimers are shown in surface representations with the monomers shown in light grey and dark grey, respectively. The differences between each pair are shown in red. The upper left and lower right triangular sections show a top and bottom view of the protein, respectively. The diagonal elements show the NS1 structure in cartoon representation. Images were generated using PyMol [21].

Figure 2 illustrates the pairwise differences visualised for the NS1 dimers of ZIKV, KUNV, YFV and DENV serotypes. The “minor degree of differences” among three ZIKV strains, as illustrated by minute amounts of red, corroborated that almost similar titres of ZIKV would exhibit reactivity, regardless of the strain. This is regardless of the Uganda strain, which has slightly faster growth kinetics than other strains. Although YFV and KUNV showed “greater differences” to DENV dimers, a high viral load would still cause cross-reactivity. Cross-referencing of differential reactivity of different assays with the investigated viruses allowed us to identify the epitopes likely to be targeted by various antibodies used in each assay. We compared the similarity of epitopes defined as a 12A region surrounding a specific residue among all viruses and correlated the identities in each epitope with the differences in reactivity of each assay to different viruses. Five distinct epitope candidates were predicted and are shown in Figure S4. Several of them were predicted to be shared between different assays. Individual assays tend to target one to two predicted epitopes. The pattern of differential reactivity between the assays corresponded to the observed sequence conservation around respective epitope/s.

## 4. Discussion

Antigenic cross-reactivity among flaviviruses has been recognised as a dire challenge for the accurate diagnosis of infections, particularly in endemic settings where multiple flaviviruses cocirculate [3]. This could be applicable to sNS1 protein, which is widely used as an early diagnostic marker for flavivirus infections such as dengue [4]. In the present study, we investigated the likelihood of detecting ZIKV and other flaviviruses when using NS1-Ag-based detection assays designed for dengue diagnostics. Our findings showed that NS1 produced by other flavivirus infections could generate false-positive signals when using DENV NS1-Ag-based rapid assays but at significantly higher virus titres than DENV. These cross-reactive virus titres were higher than those reported in natural infections. For example, the lowest ZIKV titre that gave a positive signal in the SD Bioline Dengue NS1 Ag and Panbio Dengue Early Ag rapid assays (6–7 log pfu/mL, 11–13 log RNA copies/mL) in our experiments was higher than that reported in patients. Previous reports from Indonesia, Brazil, Yap Island and Nicaragua suggest that ZIKV-infected patients generally harbour infectious viral titres (4.25 × 10^3^ pfu/mL) and RNA copy numbers in sera (< 1.12 × 10^5^ copies/mL) and urine (2.5 × 10^5^ RNA copies/mL) [17,22,23,24,25]. Even the virus load detected on the first day of a Zika fever case (7.49 log RNA copies/mL in sera and 2.5 × 10^5^ RNA copies/mL in urine) was negative for the SD BIOLINE Dengue NS1 Ag rapid test [26].

Similarly, the highest RNA load of WNV and YFV in plasma and serum of acute patients has previously been reported to be 5 × 10^4^ copies/mL [27] and 1.4 × 10^4^ copies/mL [28], respectively. These RNA loads were substantially lower than those of DENV-1–4 detectable by dengue NS1-Ag-based rapid assays (7.21–9.83 log RNA copies/mL). Moreover, the lowest reactive titres of KUNV, a subtype of WNV, and YFV that generated false-positive results with the SD Bioline Dengue NS1 Ag and Panbio Dengue Early Ag rapid assays were significantly higher than those of DENV-1–4. These observations suggest that viral loads of ZIKV, WNV and YFV present in natural infections are likely to be much lower than the threshold detectable by rapid dengue NS1 Ag detection assays. Our findings, therefore, concur with previous reports [14] that it is unlikely for commercial DENV NS1 Ag detection assays to generate false-positive test results among naturally occurring acute flavivirus infections, other than dengue, in endemic settings.

Interestingly, two DENV NS1 Ag detection ELISAs did not cross-react with other flaviviruses, except KUNV, in our experiments, demonstrating the higher specificity of ELISA compared with lateral flow formats, as shown in previous studies [20,21]. The performance of immunoassays largely depends on the quality of the antibodies used and the uniqueness of the epitopes targeted by those antibodies [29]. Less unique epitopes tend to weaken the specificity of these assays, especially when antibodies generated against antigenic domains of related pathogens demonstrate differential affinities towards targeted epitope regions. Moreover, antibodies may contain a variety of binding sites (paratopes) and seldom demonstrate polyfunctional binding [29,30] that allows undesired binding when the antibody/antigen concentrations and affinity requirements are optimal. This may explain the cross-reactivity observed among different flaviviruses in our evaluation, especially for undesired viruses at high pathogen/RNA loads. It is known that monoclonal antibodies react with different epitope regions on NS1 [31] and these regions are of variable length and amino acid composition. Since sNS1 commercial assays tend to use different antibodies that may target nonidentical epitopes, cross-reactivity can be variable among flaviviruses, as observed in the present study. The antibodies used and epitopes targeted by them in each commercial assay are proprietary. However, by comparing the similarity of residues in the central epitope region of NS1 proteins with differential reactivity of different assays, we could predict the epitopes likely to be targeted by each assay. The pattern of differential reactivity between the assays corresponded to the observed sequence conservation around the respective epitope/s. These findings suggest the possibility of the cross-reactivity of DENV NS1 Ag assays and corroborate our observations on assay performance.

Commercial Dengue NS1 Ag detection assays have also been used as a tool for the surveillance of DENV in field-caught mosquitoes. This approach has demonstrated the benefits of dengue NS1 Ag rapid assays over PCR [18,32,33]. The reported detection threshold of 10^6^ RNA copies in DENV-infected mosquitoes by dengue NS1 Ag rapid assays was consistent with our readings for DENV cultures. So far, there are no reports on the cross-reactivity of ZIKV field-caught mosquitoes to dengue NS1 Ag rapid assays. Based on our surveillance data, the local field-caught mosquitoes (*n* = 36) that tested positive for ZIKV by PCR carried an estimated viral load of ~8 log RNA copies/mL (unpublished data). This level is also below the cross-reactivity threshold shown for the rapid assays in the present study. However, testing field-caught ZIKV-positive mosquitoes in pools might concentrate viral proteins to a level detectable by dengue NS1 Ag rapid assays. Thus, it is advisable to validate positive dengue NS1 Ag rapid assay results among field-caught mosquitoes with virus-specific PCR assays.

Antigen structural distinction among flavivirus NS1 proteins has been a hurdle in assay development for point-of-care diagnostics. While the average sequence identity between ZIKV and DENV NS1 proteins is about 55%, exclusivities of ZIKV NS1 protein have been reported [34,35]. These include a negatively charged glutamate residue located at position 315 of ZIKV NS1 [34] and a unique region formed by residues 108–129 on the wing domain flexible loop of ZIKV NS1 [35]. Recently, Marjorie et al. reported the high identity of linear NS1 epitopes of DENV-2 and ZIKV, rendering them not ideal for differential diagnostics due to immunological cross-reactivity [36]. Virus-specific, surface-exposed peptides of NS1 that could distinguish different flaviviruses and subtypes have also been suggested as targets for diagnostics development [37,38]. Further efforts would be required to understand the structural folding, glycosylation and binding affinity as well as differences in lipid cargo and the consequent higher-order structure of the hexameric form of such peptides/proteins prior to generating specific immunogenic antibodies critical for the development of precise diagnostics assays.

## 5. Conclusions

Our study has highlighted that substantially high levels of ZIKV, KUNV and YFV viral loads would be required to generate false-positive results when using NS1 Ag assays designed for the diagnosis of dengue. These cross-reactive levels are generally higher than virus titres reported in natural infections due to respective flaviviruses. However, our observations are based on cell culture supernatants, which are different from actual clinical samples (serum, plasma, urine, etc). Even though the plasma viraemia and sNS1 concentrations have been shown to correlate positively over time in natural DENV infections, the magnitude of sNS1 levels tends to vary markedly by serotype and immune status [8,39]. Another limitation of our study is that though we determined the detection thresholds of DENV NS1 Ag detection assays for other flaviviruses based on quantitation of RNA copies and infective virus titres, virus-specific NS1 protein quantification assays are ideally required to measure the level of NS1 proteins in culture or clinical specimens. Such quantification assays would aid in the determination of ideal detection thresholds of assays designed for a particular flavivirus and alleviate the cross-reactivity challenges in assay development. Nevertheless, our findings support the notion that commercially available DENV NS1 assays, especially ELISAs, are generally acceptable for the diagnosis of dengue fever in endemic areas where other flaviviruses cocirculate. However, NS1 rapid assays, which can be performed in point-of-care settings with limited resources, provide a practical advantage over ELISAs. The identification of target epitopes/regions that are able to discriminate flaviviruses would be essential to put forth an ideal diagnostic tool with high specificity among different flaviviruses.

## Figures and Tables

**Table 1:** Details of virus isolates used in the study.

Virus	Isolate	GenBank Accession No.	Lineage/Genotype	Isolation Location	Source
ZIKV Uganda (ZU)	MR766	LC002520	African	Uganda, 1947	ATCC© VR-84™
ZIKV Thailand (ZT)	PLCal_ZV	KF993678	Asian	Thailand, 2013	National Microbiology Laboratory, Canada
ZIKV Puerto Rico (ZPR)	PRVABC59	KU501215	Asian	Puerto Rico, 2015	ATCC© VR-1843™
DENV-1	EHIE11986Y13	KJ806943	Genotype I	Singapore, 2013	EHI, Singapore
DENV-2	EHIE18944Y13	KR779784	Cosmopolitan Clade 1b	Singapore, 2013	EHI, Singapore
DENV-3	EHIE26592Y13	KR685235	Genotype III	Singapore, 2013	EHI, Singapore
DENV-4	EHIE2641Y08	JN019830	Genotype II	Singapore, 2008	EHI, Singapore
CHIKV	EHICH06071Y13	KP685237	Asian	Singapore, 2013	EHI, Singapore
KUNV	KUNV_EHI	MF289571	ND	ND	ND
YFV	YFV_EHI	MF289572	17D vaccine	ND	ND

All viral stocks used in the experiments were titrated by plaque assays as previously described [15]. EHI: Environmental Health Institute; ND: no data; ZIKV: Zika virus; DENV: dengue virus; CHIKV: chikungunya virus; KUNV: Kunjin virus; YFV: yellow fever virus.

**Table 2:** Lowest titres of dengue virus serotypes detected by dengue nonstructural 1 antigen (NS1 Ag) detection assays.

Dengue Virus (DENV)	Dengue NS1 Ag Rapid Assays			Dengue NS1 Ag ELISAs	
	SD BIOLINE Dengue NS1 Ag Rapid Test	Panbio Dengue Early Rapid	Bio-Rad Dengue NS1 Ag STRIP	SD Dengue NS1 Ag ELISA	Panbio ELISA
DENV-1	3.35 ± 0.09 (7.21 ± 0.24)	4.22 ± 0.14 (8.07 ± 0.07)	4.22 ± 0.14 (8.07 ± 0.07)	0 (5.41 ± 3.13)	3.25 ± 0.02 (7.00 ± 0.12)
DENV-2	3.59 ± 0.04 (7.70 ± 0.14)	4.08 ± 0.20 (8.10 ± 0.06)	3.59 ± 0.04 (7.70 ± 0.14)	0.57 ± 0.98 (2.03 ± 3.51)	3.12 ± 0.07 (7.10 ± 0.21)
DENV-3	3.07 ± 0.18 (7.58 ± 0.10)	3.39 ± 0.04 (8.15 ± 0.04)	3.07 ± 0.18 (7.58 ± 0.10)	0.57 ± 0.98 (5.40 ± 0.29)	2.20 ± 0.17 (6.64 ± 0.22)
DENV-4	3.75 ± 0.18 (9.15 ± 0.13)	4.22 ± 0.10 (9.83 ± 0.26)	2.94 ± 0.10 (8.83 ± 0.12)	0 (6.66 ± 0.15)	2.43 ± 0.23 (8.17 ± 0.02)

DENV cultures were harvested every 6 h postinfection (h.p.i.) until 60 h.p.i. and were titrated in triplicate. RNA copy numbers were also quantitated in the same triplicate samples by using a probe-based quantitative real-time PCR (qRT-PCR) assay [18]. Each supernatant was tested with DENV NS1 Ag detection assays according to the manufacturers’ instructions. The testing was done in two independent time-course experiments. The titres shown are mean values, expressed in log pfu/mL ± standard deviation and log RNA copies/mL ± standard deviation (in brackets). pfu: plaque-forming unit.

**Table 3:** Detection of KUNV, YFV and CHIKV for Dengue NS1 Ag rapid assays and Dengue NS1 ELISAs.

Virus	SD BIOLINE Dengue NS1 Ag Rapid Test	Panbio Dengue Early Rapid	Bio-Rad Dengue NS1 Ag STRIP	SD Dengue NS1 Ag ELISA	Panbio ELISA
KUNV	Reactive 5.96 ± 0.23	Reactive 6.50 ± 0.11	Nonreactive ≤7.46 ± 0.06	Nonreactive ≤7.93 ± 0.55	Reactive 5.96 ± 0.23
YFV	Reactive 7.14 ± 0.37	Nonreactive ≤7.13 ± 0.46	Nonreactive ≤7.13 ± 0.46	Nonreactive ≤7.14 ± 0.37	Nonreactive ≤7.43 ± 0.06
CHIKV	Nonreactive ≤6.98 ± 0.27	Nonreactive ≤6.98 ± 0.27	Nonreactive ≤6.98 ± 0.27	Nonreactive ≤6.98 ± 0.27	Nonreactive ≤6.98 ± 0.27

Each isolate was titrated in triplicate at each time point as described in the Material and Methods. Each supernatant was tested with DENV NS1 Ag detection assays according to the manufacturers’ instructions. The testing was done in two independent time-course experiments. The titres shown are mean values, expressed in log pfu/mL ± standard deviation. The titres shown as reactive are the lowest, whereas those shown as nonreactive are the highest. RNA copy numbers were not quantitated for KUNV, YFV and CHIKV. pfu: plaque-forming unit.

## Supplementary Materials

The following are available online at https://www.mdpi.com/2075-4418/10/1/11/s1, Figure S1: Growth kinetics of DENV serotypes. Figure S2: Growth kinetics of ZIKV. Figure S3: Growth kinetics of KUNV and YFV. Figure S4: Predicted NS1 epitopes potentially targeted by antibodies used in commercial assays. Table S1: Cross-reactive titres of ZIKV for dengue NS1 Ag detection assays.

## References

[1] Gubler D.J., Vasilakis N., Musso D. (2017). History and Emergence of Zika Virus. J. Infect. Dis..

[2] Song B.H., Yun S.I., Woolley M., Lee Y.M. (2017). Zika virus: History, epidemiology, transmission, and clinical presentation. J. Neuroimmunol..

[3] Landry M.L., St George K. (2017). Laboratory Diagnosis of Zika Virus Infection. Arch. Pathol. Lab. Med..

[4] Muller D.A., Depelsenaire A.C., Young P.R. (2017). Clinical and Laboratory Diagnosis of Dengue Virus Infection. J. Infect. Dis..

[5] Winkler G., Maxwell S.E., Ruemmler C., Stollar V. (1989). Newly synthesized dengue-2 virus nonstructural protein NS1 is a soluble protein but becomes partially hydrophobic and membrane-associated after dimerization. Virology.

[6] Flamand M., Megret F., Mathieu M., Lepault J., Rey F.A., Deubel V. (1999). Dengue virus type 1 nonstructural glycoprotein NS1 is secreted from mammalian cells as a soluble hexamer in a glycosylation-dependent fashion. J. Virol..

[7] Alcon S., Talarmin A., Debruyne M., Falconar A., Deubel V., Flamand M. (2002). Enzyme-linked immunosorbent assay specific to Dengue virus type 1 nonstructural protein NS1 reveals circulation of the antigen in the blood during the acute phase of disease in patients experiencing primary or secondary infections. J. Clin. Microbiol..

[8] Muller D.A., Young P.R. (2013). The flavivirus NS1 protein: molecular and structural biology, immunology, role in pathogenesis and application as a diagnostic biomarker. Antiviral Res..

[9] Ding X.X., Li X.F., Deng Y.Q., Guo Y.H., Hao W., Che X.Y., Qin C.F., Fu N. (2014). Development of a double antibody sandwich ELISA for West Nile virus detection using monoclonal antibodies against non-structural protein 1. PLoS ONE.

[10] Alcon-LePoder S., Sivard P., Drouet M.T., Talarmin A., Rice C., Flamand M. (2006). Secretion of flaviviral non-structural protein NS1: from diagnosis to pathogenesis. Novartis Found. Symp.

[11] Kumar J.S., Parida M., Rao P.V. (2011). Monoclonal antibody-based antigen capture immunoassay for detection of circulating non-structural protein NS1: implications for early diagnosis of Japanese encephalitis virus infection. J. Med. Virol..

[12] Wee S., Alli-Shaik A., Kek R., Swa H.L.F., Tien W.P., Lim V.W., Leo Y.S., Ng L.C., Hapuarachchi H.C., Gunaratne J. (2019). Multiplex targeted mass spectrometry assay for one-shot flavivirus diagnosis. Proc. Natl. Acad. Sci. USA.

[13] Gyurech D., Schilling J., Schmidt-Chanasit J., Cassinotti P., Kaeppeli F., Dobec M. (2016). False positive dengue NS1 antigen test in a traveller with an acute Zika virus infection imported into Switzerland. Swiss Med. Wkly..

[14] Matheus S., Boukhari R., Labeau B., Ernault V., Bremand L., Kazanji M., Rousset D. (2016). Specificity of Dengue NS1 Antigen in Differential Diagnosis of Dengue and Zika Virus Infection. Emerg. Infect. Dis..

[15] Tan L.K., Lam S., Low S.L., Tan F.H., Ng L.C., Teo D. (2013). Evaluation of Pathogen Reduction Systems to Inactivate Dengue and Chikungunya Viruses in Apheresis Platelets Suspended in Plasma. Adv. Infect. Dis..

[16] Zhang L., Wei Q., Mao L., Liu W., Mills G.B., Coombes K. (2009). Serial dilution curve: a new method for analysis of reverse phase protein array data. Bioinformatics.

[17] Lanciotti R.S., Kosoy O.L., Laven J.J., Velez J.O., Lambert A.J., Johnson A.J., Stanfield S.M., Duffy M.R. (2008). Genetic and serologic properties of Zika virus associated with an epidemic, Yap State, Micronesia, 2007. Emerg. Infect. Dis..

[18] Tan C.H., Wong P.S., Li M.Z., Vythilingam I., Ng L.C. (2011). Evaluation of the Dengue NS1 Ag Strip(R) for detection of dengue virus antigen in Aedes aegypti (Diptera: Culicidae). Vector Borne Zoonotic Dis..

[19] Katoh K., Misawa K., Kuma K., Miyata T. (2002). MAFFT: A novel method for rapid multiple sequence alignment based on fast Fourier transform. Nucleic Acids Res..

[20] Brown W.C., Akey D.L., Konwerski J.R., Tarrasch J.T., Skiniotis G., Kuhn R.J., Smith J.L. (2016). Extended surface for membrane association in Zika virus NS1 structure. Nat. Struct Mol. Biol..

[21] Grell L., Parkin C., Slatest L., Craig P.A. (2006). EZ-Viz, a tool for simplifying molecular viewing in PyMOL. Biochem. Mol. Biol. Educ.

[22] Perkasa A., Yudhaputri F., Haryanto S., Hayati R.F., Ma’roef C.N., Antonjaya U., Yohan B., Myint K.S., Ledermann J.P., Rosenberg R. (2016). Isolation of Zika Virus from Febrile Patient, Indonesia. Emerg. Infect. Dis..

[23] Bonaldo M.C., Ribeiro I.P., Lima N.S., Dos Santos A.A., Menezes L.S., da Cruz S.O., de Mello I.S., Furtado N.D., de Moura E.E., Damasceno L. (2016). Isolation of Infective Zika Virus from Urine and Saliva of Patients in Brazil. PLoS Negl. Trop. Dis..

[24] Pessôa R., Patriota J.V., Lourdes de Souza M., Felix A.C., Mamede N., Sanabani S.S. (2016). Investigation Into an Outbreak of Dengue-like Illness in Pernambuco, Brazil, Revealed a Cocirculation of Zika, Chikungunya, and Dengue Virus Type 1. Medicine (Baltimore).

[25] Waggoner J.J., Gresh L., Vargas M.J., Ballesteros G., Tellez Y., Soda K.J., Sahoo M.K., Nuñez A., Balmaseda A., Harris E. (2016). Viremia and Clinical Presentation in Nicaraguan Patients Infected with Zika Virus, Chikungunya Virus, and Dengue Virus. Clin. Infect. Dis..

[26] Tan C.H., Tan L.K., Hapuarachchi H.C., Lai Y.L., Wong P.S.J., Yap G., Mak K.W., Wong W.Y., Leo Y.S., Wong M.C. (2018). Viral and Antibody Kinetics, and Mosquito Infectivity of an Imported Case of Zika Fever Due to Asian Genotype (American Strain) in Singapore. Viruses.

[27] Barzon L., Pacenti M., Franchin E., Squarzon L., Sinigaglia A., Ulbert S., Cusinato R., Palù G. (2014). Isolation of West Nile virus from urine samples of patients with acute infection. J. Clin. Microbiol..

[28] Chen Z., Liu L., Lv Y., Zhang W., Li J., Zhang Y., Di T., Zhang S., Liu J., Qu J. (2016). A fatal yellow fever virus infection in China: description and lessons. Emerg. Microbes Infect..

[29] Scott M.G. (1985). Monoclonal antibodies—Approaching adolescence in diagnostic immunoassays. Trends Biotechnol..

[30] Frank S.A., Frank S.A. (2002). Molecular processes: Specificity and cross-reactivity. Immunology and Evolution of Infectious Disease.

[31] Rocha L.B., Alves R.P.D.S., Caetano B.A., Pereira L.R., Mitsunari T., Amorim J.H., Polatto J.M., Botosso V.F., Gallina N.M.F., Palacios R. (2017). Epitope Sequences in Dengue Virus NS1 Protein Identified by Monoclonal Antibodies. Antibodies (Basel).

[32] Andries A.C., Duong V., Ngan C., Ong S., Huy R., Sroin K.K., Te V., Y B., Try P.L., Buchy P. (2012). Field evaluation and impact on clinical management of a rapid diagnostic kit that detects dengue NS1, IgM and IgG. PLoS Negl. Trop. Dis..

[33] Lau S.M., Chua T.H., Sulaiman W.Y., Joanne S., Lim Y.A., Sekaran S.D., Chinna K., Venugopalan B., Vythilingam I. (2017). A new paradigm for Aedes spp. surveillance using gravid ovipositing sticky trap and NS1 antigen test kit. Parasit Vectors.

[34] Stettler K., Beltramello M., Espinosa D.A., Graham V., Cassotta A., Bianchi S., Vanzetta F., Minola A., Jaconi S., Mele F. (2016). Specificity, cross-reactivity, and function of antibodies elicited by Zika virus infection. Science.

[35] Song H., Qi J., Haywood J., Shi Y., Gao G.F. (2016). Zika virus NS1 structure reveals diversity of electrostatic surfaces among flaviviruses. Nat. Struct Mol. Biol..

[36] Freire M., Pol-Fachin L., Coelho D.F., Viana I.F.T., Magalhaes T., Cordeiro M.T., Fischer N., Loeffler F.F., Jaenisch T., Franca R.F. (2017). Mapping Putative B-Cell Zika Virus NS1 Epitopes Provides Molecular Basis for Anti-NS1 Antibody Discrimination between Zika and Dengue Viruses. ACS Omega.

[37] Lee A.J., Bhattacharya R., Scheuermann R.H., Pickett B.E. (2017). Identification of diagnostic peptide regions that distinguish Zika virus from related mosquito-borne Flaviviruses. PLoS ONE.

[38] Chang H.H., Huber R.G., Bond P.J., Grad Y.H., Camerini D., Maurer-Stroh S., Lipsitch M. (2017). Systematic analysis of protein identity between Zika virus and other arthropod-borne viruses. Bull. World Health Organ..

[39] Duyen H.T., Ngoc T.V., Ha do T., Hang V.T., Kieu N.T., Young P.R., Farrar J.J., Simmons C.P., Wolbers M., Wills B.A. (2011). Kinetics of plasma viremia and soluble nonstructural protein 1 concentrations in dengue: differential effects according to serotype and immune status. J. Infect. Dis..

