# Supplementary material for: Flavivirus Cross-Reactivity to Dengue Nonstructural Protein 1 Antigen Detection Assays
Source: Diagnostics (Basel). 2019 Dec 24;10(1):11. doi: 10.3390/diagnostics10010011 (PMC7167843; doi:10.3390/diagnostics10010011)
Supplement: Supplementary file 1 [file diagnostics-10-00011-s001.pdf]

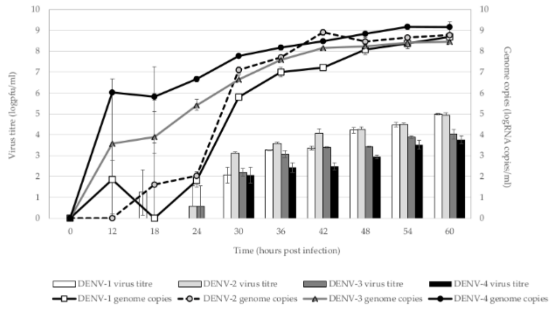

Figure S1. Growth kinetics of DENV serotypes.

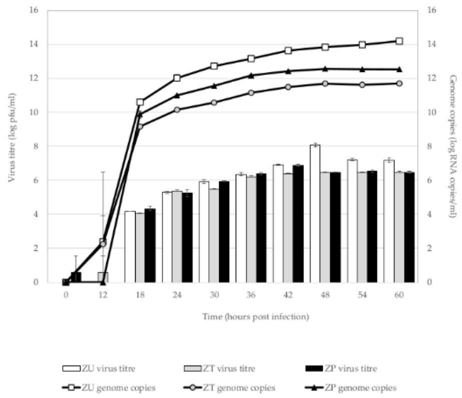

Figure S2: Growth kinetics of ZIKV.

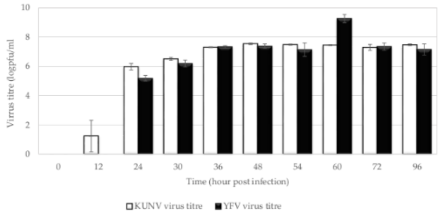

Figure S3: Growth kinetics of KUNV and YFV. Figure S4: Predicted NS1 epitopes potentially targetted by antibodies used in commercial assays.

Table S1: Cross-reactive titres of Zika virus (ZIKV) for dengue NS1 Ag detection assays.

Virus strain	Dengue NS1 Ag Rapid assays			Dengue NS1 Ag ELISA assays	
	SD BIOLINE Dengue NS1 Ag rapid test	Panbio Dengue Early Rapid	Biorad Dengue NS1 Ag STRIP	SD Dengue NS1 Ag ELISA	PanBio ELISA
ZIKV Uganda (ZU)	Reactive<br>6.36 ± 0.08<br>(13.15 ± 0.01)	Reactive<br>6.92 ± 0.04<br>(13.62 ± 0.03)	Non-reactive<br>≤ 7.21 ± 0.08<br>(≤ 13.97 ± 0.13)	Non-reactive<br>≤ 7.21 ± 0.12<br>(≤13.97 ± 0.13)	
ZIKV Thailand (ZT)	Reactive<br>6.21 ± 0.06<br>(11.14 ± 0.06)	Reactive<br>6.37 ± 0.06<br>(11.48 ± 0.04)	Non-reactive<br>≤ 6.46 ± 0.02<br>(≤ 11.61 ± 0.04)	Non-reactive<br>≤ 6.46 ± 0.02<br>(≤ 11.61 ± 0.04)	

ZIKV Puerto Rico (ZP)	Reactive<br>6.38 ± 0.07<br>(12.17 ± 0.03)	Reactive<br>6.88 ± 0.06<br>(12.41 ± 0.06)	Non-reactive<br>≤ 6.52 ± 0.08<br>(≤ 12.52 ± 0.05)	Non-reactive<br>≤ 6.52 ± 0.08<br>(≤12.52 ± 0.05)

ZIKV culture supernatants were harvested at every 6-12-hours post-infection (h.p.i.) till 60 h.p.i., and tested with DENV NS1 Ag detection assays according to the manufacturers' instructions. The viral load of culture supernatants first tested either positive (reactive) or negative (non-reactive to cultures collected till 60 h.p.i.) in each assay is given. The titres are given in log pfu/mL ± standard deviation. Those in brackets are log RNA copy/mL ± standard deviation. pfu: plaque forming unit.
